# (*E*)-1-(2,6-Di­chloro­phen­yl)-2-(3-nitro­benzyl­idene)hydrazine: crystal structure and Hirshfeld surface analysis

**DOI:** 10.1107/S2056989020009433

**Published:** 2020-07-17

**Authors:** Zeliha Atioğlu, Mehmet Akkurt, Namiq Q. Shikhaliyev, Gulnar T. Suleymanova, Gulnare V. Babayeva, Nurana V. Gurbanova, Gunay Z. Mammadova, Sixberth Mlowe

**Affiliations:** a İlke Education and Health Foundation, Cappadocia University, Cappadocia Vocational College, The Medical Imaging Techniques Program, 50420 Mustafapaşa, Ürgüp, Nevşehir, Turkey; b Department of Physics, Faculty of Sciences, Erciyes University, 38039 Kayseri, Turkey; c Organic Chemistry Department, Baku State University, Z. Khalilov str. 23, AZ, 1148 Baku, Azerbaijan; d University of Dar es Salaam, Dar es Salaam University College of Education, Department of Chemistry, PO Box 2329, Dar es Salaam, Tanzania

**Keywords:** crystal structure, isomer, 2,6-di­chloro­phenyl ring, 3-nitro­benzyl­idene ring, Hirshfeld surface analysis

## Abstract

The stabilized conformation of the title compound, C_13_H_9_Cl_2_N_3_O_2_, is similar to that of the isomeric compound (*E*)-1-(2,6-di­chloro­phen­yl)-2-(2-nitro­benzyl­idene)hydrazine. The 2,6-di­chloro­phenyl ring and the nitro-substituted benzene ring form a dihedral angle of 26.25 (16)°. In the crystal, face-to-face π-π stacking inter­actions along the *a*-axis direction occur between the centroids of the 2,6-di­chloro­phenyl ring and the nitro-substituted benzene ring. The mol­ecules are further linked by C—H⋯O contacts and N—H⋯O and C—H⋯Cl hydrogen bonds, forming pairs of hydrogen-bonded mol­ecular layers parallel to (100). The Hirshfeld surface analysis of the crystal structure indicates that the most important contributions to the crystal packing are from H⋯H (22.1%), Cl⋯H/H⋯Cl (20.5%), O⋯H/H⋯O (19.7%), C⋯C (11.1%) and C⋯H/H⋯C (8.3%) inter­actions.

## Abstract 2

In the crystal, face-to-face π-π stacking inter­actions along the *a*-axis direction occur between the 2,6-di­chloro­phenyl ring and nitro-substituted benzene ring of the title mol­ecule. The mol­ecules are further linked by C—H⋯O contacts and N—H⋯O and C—H⋯Cl hydrogen bonds, forming pairs of hydrogen-bonded mol­ecular layers parallel to (100).

## Chemical context   

Schiff bases as well as hydrazone ligands and their complexes have attracted much attention because of their high synthetic potential for organic and inorganic chemistry and their diverse useful properties (Maharramov *et al.*, 2018 ▸; Mahmudov *et al.*, 2014 ▸). The analytical and catalytic properties of this class of compounds are strongly dependent on the groups attached to the hydrazone moiety (Shixaliyev *et al.*, 2019 ▸). On the other hand, inter­molecular inter­actions organize mol­ecular architectures, which play a critical role in synthesis, catalysis, micellization, *etc* (Akbari *et al.*, 2017 ▸; Gurbanov *et al.*, 2018 ▸; Mahmoudi *et al.*, 2018 ▸ and references cited therein). New types of weak inter­actions such as halogen, chalcogen, pnictogen and tetrel bonds or their cooperation with hydrogen bonds are able to drive the synthesis and catalysis, as well as improve properties of materials (Mizar *et al.*, 2012 ▸; Mahmudov *et al.*, 2019 ▸ and references cited therein). For that, the main skeleton of the aryl­hydrazone ligand should be extended with weak bond-donor centre(s). In order to continue our work in this perspective, we have functionalized a new azo dye, (*E*)-1-(2,6-di­chloro­phen­yl)-2-(3-nitro­benzyl­idene)hydrazine, (I), which provides inter­molecular non-covalent inter­actions.
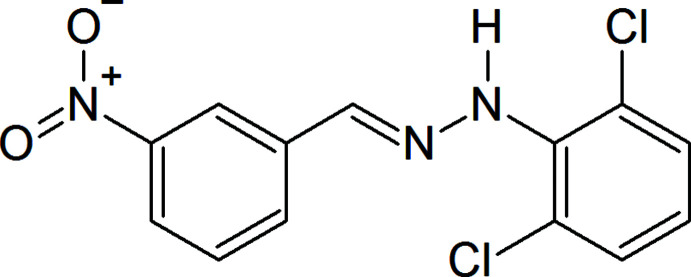


## Structural commentary   

**Figure 1:**
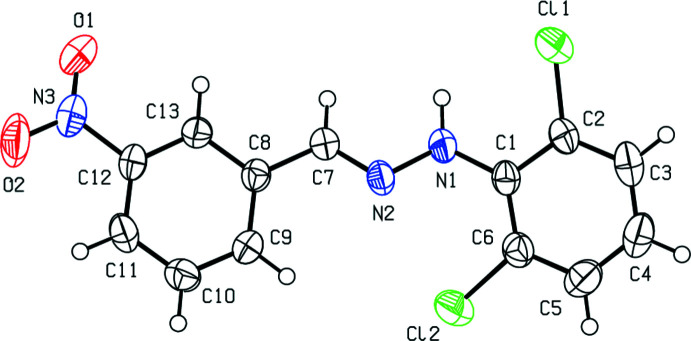
The mol­ecular structure of the title compound, showing the atom labelling and displacement ellipsoids drawn at the 50% probability level.

**Figure 2:**
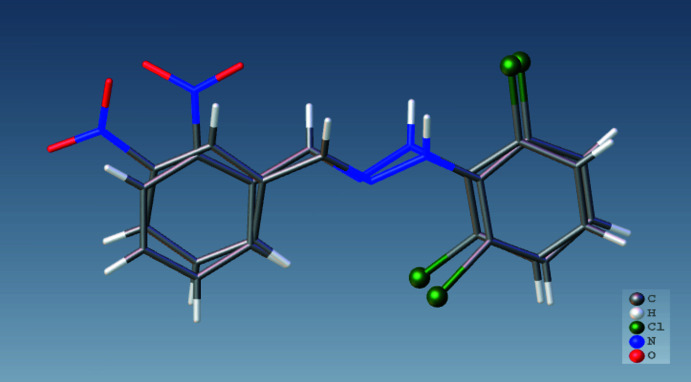
Overlay of the title compound and the isomer *(E)*-1-(2,6-di­chloro­phen­yl)-2-(2-nitro­benzyl­idene)hydrazine (Çelikesir *et al.*, 2020 ▸).

The title mol­ecule (Fig. 1 ▸) has an *E* configuration about the C=N bond. The 2,6-di­chloro­phenyl ring and the nitro-substituted benzene ring of the title compound are inclined at 26.25 (16)°, while the nitro group is skewed out of the attached benzene ring plane by 6.3 (2)°. The conformation is stabilized by an intra­molecular N1—H1*N*⋯Cl1 inter­action, which forms an *S*(6) graph-set motif (Table 1 ▸). The conformation of the title compound can be compared with that of the isomeric compound (*E*)-1-(2,6-di­chloro­phen­yl)-2-(2-nitro­benzyl­idene)hydrazine (CSD refcode KUWGOB; Çelikesir *et al.*, 2020 ▸). Fig. 2 ▸ shows the overlay of the two isomers. The r.m.s. deviation of the overlay between the two isomers is 0.003 Å. In the 2-nitro isomer, the dihedral angles are 21.16 (14) (between the phenyl rings) and 27.06 (18)° (between between the nitro group and the phenyl ring). The difference in angles may be due to the steric inter­action resulting from the position of the nitro group on the benzene ring to which it is attached. The Cl1—C2—C1—N1, Cl2—C6—C1—N1, C2—C1—N1—N2, C1—N1—N2—C7, N1—N2—C7—C8, N2—C7—C8—C13, N2—C7—C8—C9, C8—C13—C12—N3, C13—C12—N3—O1 and C13—C12—N3—O2 torsion angles are −0.1 (4), 3.4 (4), −147.1 (3), 177.3 (3), 178.2 (2), 165.5 (3), −15.6 (4), 178.8 (3), 6.2 (4) and −174.4 (3) °, respectively.

## Supra­molecular features   

**Figure 3:**
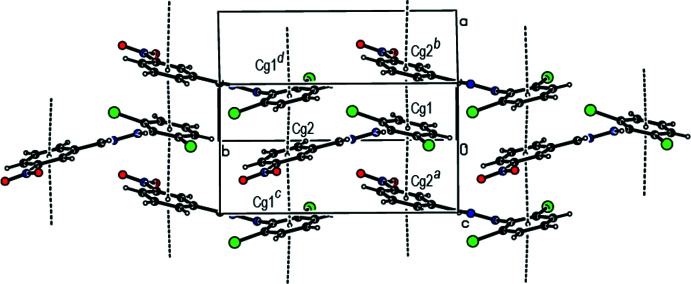
A view of the π–π stacking inter­actions of the title compound. *Cg*1 and *Cg*2 are the centroids of the C1–C6 and C8–C13 benzene rings, respectively. [Symmetry codes: (*a*) −*x*, −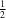 + *y*, 1 − *z*; (*b*) 1 − *x*, −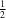 + *y*, 1 − *z*; (*c*) −*x*, 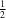 + *y*, 1 − *z*; (*d*) 1 − *x*, 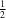 + *y*, 1 − *z*].

**Figure 4:**
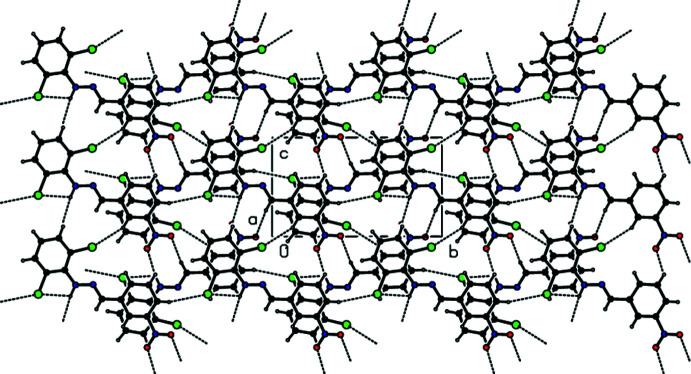
A general view of the crystal packing of the title compound along the *a* axis with hydrogen bonds and contacts shown as dashed lines.

In the crystal, face-to-face π–π stacking inter­actions [*Cg*1⋯*Cg*2(−*x*, −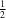 + *y*, 1 − *z*) = 3.753 (2) Å with slippage of 1.380 Å and *Cg*l⋯*Cg*2(1 − *x*, −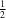 + *y*, 1 − *z*) = 3.761 (2) Å with slippage of 1.423 Å, where *Cg*1 and *Cg*2 are the centroids of the C1–C6 and C8–C13 rings, respectively] occur between the 2,6-di­chloro­phenyl ring and the nitro-substituted benzene ring of the title mol­ecule along the *a*-axis direction (Fig. 3 ▸). The mol­ecules are further linked by C—H⋯O contacts and N—H⋯O and C—H⋯Cl hydrogen bonds, forming pairs of hydrogen-bonded mol­ecular layers parallel to (100) (Tables 1 ▸ and 2 ▸; Figs. 4 ▸ and 5 ▸). In the crystal, a C—Cl⋯π inter­action is also observed [C2—Cl1⋯*Cg*2 (−*x*, −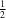 + *y*, 1 − *z*) = 3.9373 (18) Å, C2—Cl1⋯*Cg*2 = 62.59 (10)°, where *Cg*2 is the centroid of the C8–C13 ring]. The large Cl⋯*Cg*2 distance and acute C–Cl⋯*Cg*2 angle, however, indicate that this inter­action is only weak.

## Hirshfeld surface analysis   

**Figure 6:**
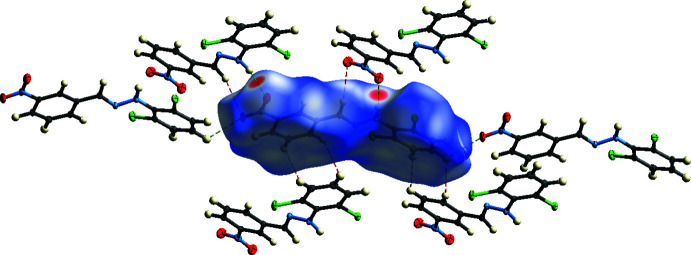
A view of the Hirshfeld surface mapped over *d*_norm_ in the range −0.2694 to 1.2224 arbitrary units showing C—H⋯O, N—H⋯O hydrogen bonds and H⋯H inter­actions (van der Waals inter­actions). Applied colours for atoms: grey = C, white = H, blue = N, red = O and green = Cl.

**Figure 7:**
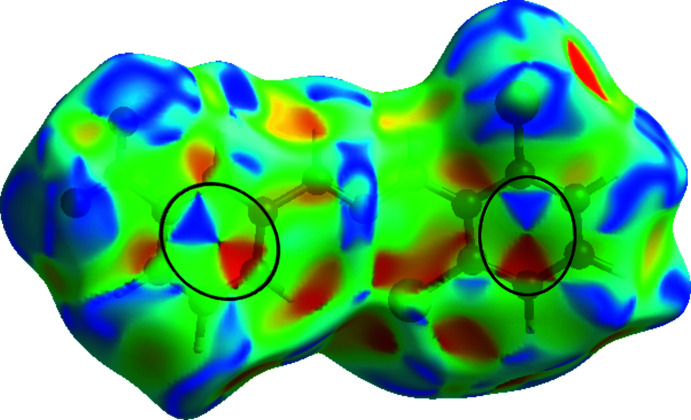
View of the three-dimensional Hirshfeld surfaces of the title compound plotted over shape-index.

The Hirshfeld surface analysis (Spackman & Jayatilaka, 2009 ▸) and the associated two-dimensional fingerprint plots (McKinnon, *et al.*, 2007 ▸) were performed with *Crystal Explorer17* (Turner *et al.*, 2017 ▸) to investigate the inter­molecular inter­actions and surface morphology. The Hirshfeld surface mapped over *d*_norm_ in the range −0.2694 to 1.2224 a.u. and corresponding colours from red (shorter distance than the sum of van der Waals radii) over white to blue (longer distance than the sum of van der Waals radii) is shown in Fig. 6 ▸. The red points, which represent closer contacts and negative *d*_norm_ values on the surface, correspond to the N—H⋯O, C—H⋯O and C—H⋯Cl inter­actions (Table 2 ▸). The shape-index of the Hirshfeld surface is a tool for visualizing the π–π stacking by the presence of adjacent red and blue triangles. The plot of the Hirshfeld surface mapped over shape-index shown in Fig. 7 ▸ clearly suggests that there are π–π inter­actions in the title compound.

**Figure 8:**
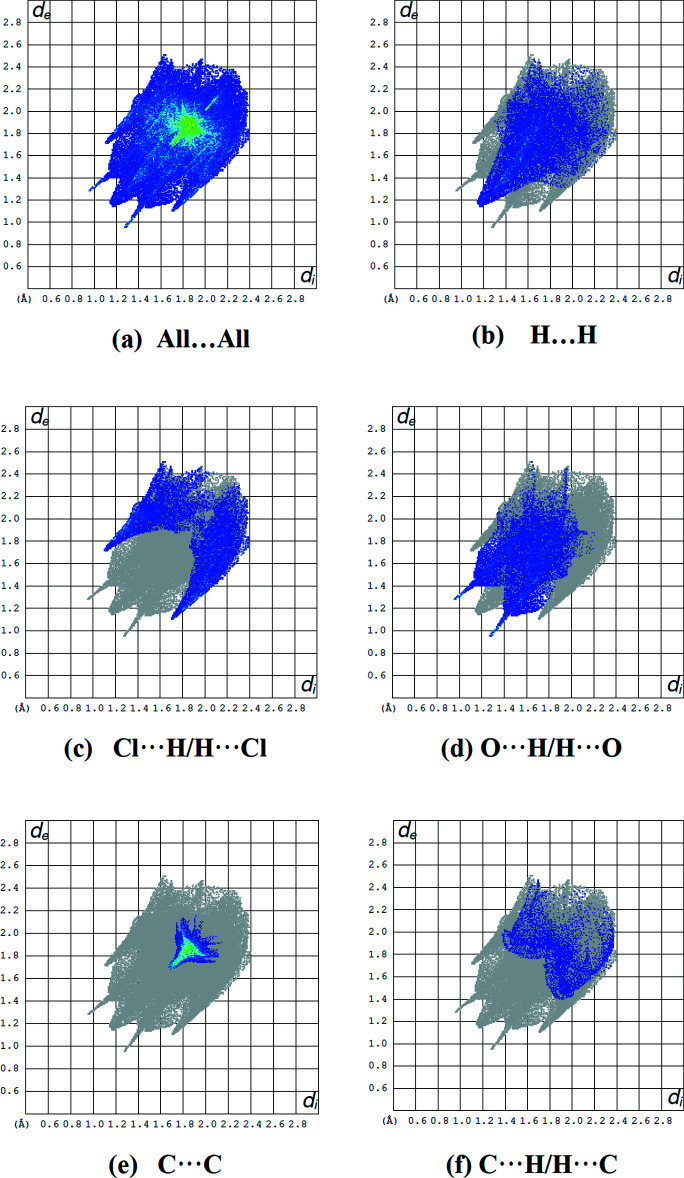
(*a*) The full two-dimensional fingerprint plot for the title compound and (*b*)–(*f*) those delineated into H⋯H (22.1%), Cl⋯H/H⋯Cl (20.5%), O⋯H/H⋯O (19.7%), C⋯C (11.1%) and C⋯H/H⋯C (8.3%) contacts, respectively.

In the crystal there are four major types of inter­action (H⋯H = 22.1%, Cl⋯H = 20.5%, O⋯H = 19.7%, C⋯C = 11.1%) on the *d*_norm_ surface. The two-dimensional fingerprint plots are shown in Fig. 8 ▸. The inter­action sequence of *d*_norm_ on the two-dimensional fingerprint plot (H⋯H) > (Cl⋯H) > (O⋯H) > (C⋯C) represents the nature of the packing in the crystal structure. The contribution of these major inter­actions governs the overall packing of crystal structure. The percentage contributions of other weak inter­actions are: C⋯H/H⋯C (8.3%), N⋯H/H⋯N (4.9%), Cl⋯C/C⋯Cl (3.3%), N⋯C/C⋯N (2.9%), Cl⋯O/O⋯Cl (2.6%), Cl⋯N/N⋯Cl (1.8%), C⋯O/O⋯C (1.7%) and Cl⋯Cl (1.2%).

## Database survey   

A search of the Cambridge Structural Database (CSD version 5.40, update of September 2019; Groom *et al.*, 2016 ▸) gave only seven entries closely resembling the title compound. Our recently published compound (*E*)-1-(2,6-di­chloro­phen­yl)-2-(2-nitro­benzyl­idene)hydrazine (KUWGOB: Çelikesir *et al.*, 2020 ▸) is an isomer of the title compound. The other six compounds are 1-(2,4-di­nitro­phen­yl)-2-[(*E*)-(3,4,5-tri­meth­oxy­benzyl­idene)hydrazine] (GISJAV: Chantrapromma *et al.*, 2014 ▸), (*E*)-1-(2,4-di­nitro­phen­yl)-2-[1-(3-meth­oxy­phen­yl)eth­yl­idene]hydrazine (XEBCEO: Fun *et al.*, 2012 ▸), 1-(2,4-di­nitro­phen­yl)-2-[(*E*)-2,4,5-tri­meth­oxy­benzyl­idene]hydrazine (AFUSEB: Fun *et al.*, 2013 ▸), (*E*)-1-(2,4-di­nitro­phen­yl)-2-(1-(2-meth­oxy­phen­yl)ethyl­idene)hydrazine (OBUJAY: Fun *et al.*, 2011 ▸), (*E*)-1-(2,4-di­nitro­phen­yl)-2-[1-(3-fluoro­phen­yl)ethyl­idene]hydrazine (PAVKAA: Chantrapromma *et al.*, 2012 ▸) and (*E*)-1-(2,4-di­nitro­phen­yl)-2-[1-(2-nitro­phen­yl)ethyl­idene]hydrazine (YAHRUW: Nilwanna *et al.*, 2011 ▸). All bond lengths (Allen *et al.*, 1987 ▸) and angles for the title compound and these related compounds are comparable and within normal ranges.

## Synthesis and crystallization   

The title compound was synthesized according to the reported method (Atioğlu *et al.*, 2019 ▸; Maharramov *et al.*, 2018 ▸). A mixture of 3-nitro­benzaldehyde (10 mmol), CH_3_COONa (0.82 g), ethanol (50 mL) and (2,6-di­chloro­phen­yl)hydrazine (10.2 mmol) was refluxed at 353 K with stirring for 2 h. The reaction mixture was cooled to room temperature and water (50 mL) was added to give a precipitate of the crude product, which was filtered off, washed with diluted ethanol (1:1 with water) and dried *in vacuo* of rotary evaporator. Crystals suitable for X-ray analysis were obtained by slow evaporation of an ethanol solution; yellow solid; yield 95%; m.p. 423 K. Analysis calculated for C_13_H_9_Cl_2_N_3_O_2_ (*M =* 310.13): C 50.35, H 2.93, N 13.55; found: C 50.32, H 2.90, N 13.47%. ^1^H NMR (300 MHz, DMSO-*d*_6_): *δ* 9.95 (1H, –NH), 8.40 (1H, –CH), 7.00–8.20 (7H, aromatic). ^13^C NMR (75 MHz, DMSO-*d*_6_): *δ* 149.00, 138.01, 137.50, 136.05, 132.11, 130.50, 129.04, 128.23, 126.24, 123.16, 119.90. ESI-MS: *m*/*z*: 311.14 [*M*+H]^+^.

## Refinement   

Crystal data, data collection and structure refinement details are summarized in Table 3 ▸. All H atoms were refined using a riding model with *d*(C—H) = 0.93 Å, *d*(N—H) = 0.95 Å and *U*_iso_ = 1.2*U*_eq_(N,C).

## Supplementary Material

Crystal structure: contains datablock(s) I. DOI: 10.1107/S2056989020009433/vm2237sup1.cif

Structure factors: contains datablock(s) I. DOI: 10.1107/S2056989020009433/vm2237Isup2.hkl

Supporting information file. DOI: 10.1107/S2056989020009433/vm2237Isup3.cml

CCDC reference: 2015528

Additional supporting information:  crystallographic information; 3D view; checkCIF report

## Figures and Tables

**Figure 5:**
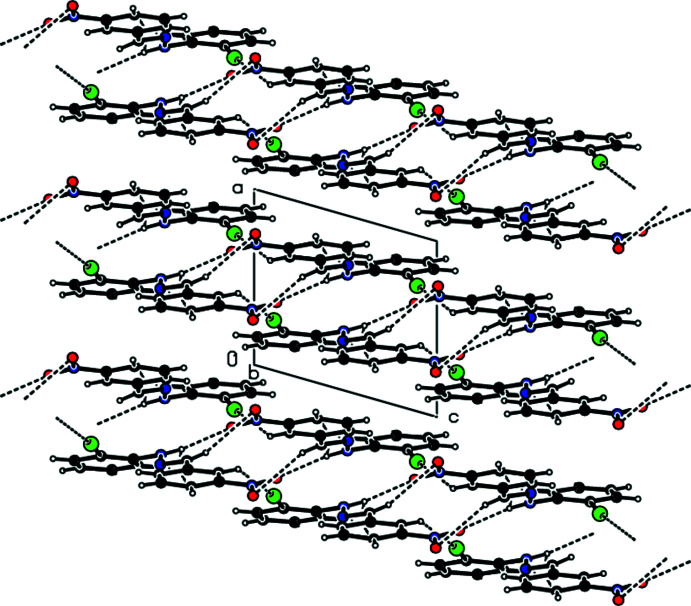
A general view of the crystal packing of the title compound along the *b* axis showing the pairs of hydrogen-bonded mol­ecular layers parallel to (100).

**Table 1:** Hydrogen-bond geometry (Å, °)

*D*—H⋯*A*	*D*—H	H⋯*A*	*D*⋯*A*	*D*—H⋯*A*
N1—H1*N*⋯Cl1	0.95	2.54	2.940 (2)	106
N1—H1*N*⋯O1^i^	0.95	2.31	3.243 (3)	168

Symmetry code: (i) 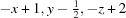.

**Table 2:** Summary of short inter­atomic contacts (Å) in the title compound

Contact	Distance	Symmetry operation
Cl1⋯H11*A*	3.13	*x*, − 1 + *y*, *z*
H1*N*⋯O1	2.31	1 − *x*, − 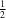 + *y*, 2 − *z*
H7*A*⋯O2	2.77	1 − *x*, − 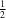 + *y*, 2 − *z*
C1⋯C11	3.405	−*x*, − 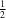 + *y*, 1 − *z*
Cl2⋯H13*A*	2.95	*x*, *y*, −1 + *z*
O2⋯H4*A*	2.66	*x*, 1 + *y*, 1 + *z*
C2⋯C8	3.426	1 − *x*, − 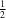 + *y*, 1 − *z*
H5*A*⋯H10*A*	2.55	−*x*, − 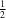 + *y*, −*z*

**Table 3:** Experimental details

Crystal data	
Chemical formula	C_13_H_9_Cl_2_N_3_O_2_
*M*_r_	310.13
Crystal system, space group	Monoclinic, *P*2_1_
Temperature (K)	296
*a*, *b*, *c* (Å)	7.1212 (14), 12.711 (3), 7.6991 (16)
β (°)	105.940 (7)
*V* (Å^3^)	670.1 (2)
*Z*	2
Radiation type	Mo *K*α
μ (mm^−1^)	0.49
Crystal size (mm)	0.26 × 0.22 × 0.18

Data collection	
Diffractometer	Bruker APEXII CCD
Absorption correction	Multi-scan (*SADABS*; Bruker, 2003 ▸)
*T*_min_, *T*_max_	0.873, 0.902
No. of measured, independent and observed [*I* > 2σ(*I*)] reflections	22230, 2744, 2392
*R*_int_	0.062
(sin θ/λ)_max_ (Å^−1^)	0.627

Refinement	
*R*[*F* ^2^ > 2σ(*F* ^2^)], *wR*(*F* ^2^), *S*	0.031, 0.064, 1.11
No. of reflections	2744
No. of parameters	181
No. of restraints	1
H-atom treatment	H-atom parameters constrained
Δρ_max_, Δρ_min_ (e Å^−3^)	0.15, −0.16
Absolute structure	Flack *x* determined using 1032 quotients [(*I* ^+^)−(*I* ^−^)]/[(*I* ^+^)+(*I* ^−^)] (Parsons *et al.*, 2013 ▸)
Absolute structure parameter	0.04 (3)

Computer programs: *APEX3* (Bruker, 2007 ▸), *SAINT* (Bruker, 2007 ▸), *SHELXT2016/6* (Sheldrick, 2015*a* ▸), *SHELXL2016/6* (Sheldrick, 2015*b* ▸), *ORTEP-3 for Windows* (Farrugia, 2012 ▸) and *OLEX2* (Dolomanov *et al.*, 2009 ▸), *PLATON* (Spek, 2020 ▸).

## supplementary crystallographic information

### Crystal data

**Table:**

C_13_H_9_Cl_2_N_3_O_2_	*F*(000) = 316
*M**_r_* = 310.13	*D*_x_ = 1.537 Mg m^−^^3^
Monoclinic, *P*2_1_	Mo *K*α radiation, λ = 0.71073 Å
*a* = 7.1212 (14) Å	Cell parameters from 9800 reflections
*b* = 12.711 (3) Å	θ = 2.8–26.3°
*c* = 7.6991 (16) Å	µ = 0.49 mm^−^^1^
β = 105.940 (7)°	*T* = 296 K
*V* = 670.1 (2) Å^3^	Plate, orange
*Z* = 2	0.26 × 0.22 × 0.18 mm

### Data collection

**Table:**

Bruker APEXII CCD diffractometer	2392 reflections with *I* > 2σ(*I*)
φ and ω scans	*R*_int_ = 0.062
Absorption correction: multi-scan (SADABS; Bruker, 2003)	θ_max_ = 26.4°, θ_min_ = 2.8°
*T*_min_ = 0.873, *T*_max_ = 0.902	*h* = −8→8
22230 measured reflections	*k* = −15→15
2744 independent reflections	*l* = −9→9

### Refinement

**Table:**

Refinement on *F*^2^	Secondary atom site location: difference Fourier map
Least-squares matrix: full	Hydrogen site location: mixed
*R*[*F*^2^ > 2σ(*F*^2^)] = 0.031	H-atom parameters constrained
*wR*(*F*^2^) = 0.064	*w* = 1/[σ^2^(*F*_o_^2^) + (0.0217*P*)^2^ + 0.0759*P*] where *P* = (*F*_o_^2^ + 2*F*_c_^2^)/3
*S* = 1.11	(Δ/σ)_max_ < 0.001
2744 reflections	Δρ_max_ = 0.15 e Å^−^^3^
181 parameters	Δρ_min_ = −0.16 e Å^−^^3^
1 restraint	Absolute structure: Flack *x* determined using 1032 quotients [(*I*^+^)-(*I*^-^)]/[(*I*^+^)+(*I*^-^)] (Parsons *et al.*, 2013)
Primary atom site location: structure-invariant direct methods	Absolute structure parameter: 0.04 (3)

### Special details

**Table:**

Geometry. All esds (except the esd in the dihedral angle between two l.s. planes) are estimated using the full covariance matrix. The cell esds are taken into account individually in the estimation of esds in distances, angles and torsion angles; correlations between esds in cell parameters are only used when they are defined by crystal symmetry. An approximate (isotropic) treatment of cell esds is used for estimating esds involving l.s. planes.

### Fractional atomic coordinates and isotropic or equivalent isotropic displacement parameters (Å^2^)

**Table:**

	*x*	*y*	*z*	*U*_iso_*/*U*_eq_	
Cl1	0.28649 (13)	0.12020 (5)	0.58310 (10)	0.0583 (2)	
Cl2	0.28511 (15)	0.43809 (7)	0.10800 (12)	0.0666 (3)	
O1	0.3919 (5)	0.7625 (2)	1.1215 (3)	0.0799 (8)	
O2	0.2541 (5)	0.9024 (2)	0.9937 (4)	0.0848 (9)	
N1	0.3362 (4)	0.34075 (17)	0.4897 (3)	0.0465 (6)	
H1N	0.401372	0.310054	0.602708	0.056*	
N2	0.2806 (3)	0.44380 (18)	0.4863 (3)	0.0397 (5)	
N3	0.3101 (4)	0.8121 (2)	0.9871 (4)	0.0531 (7)	
C1	0.2672 (4)	0.2769 (2)	0.3396 (4)	0.0365 (6)	
C2	0.2365 (4)	0.1696 (2)	0.3645 (4)	0.0411 (7)	
C3	0.1710 (5)	0.1009 (2)	0.2220 (4)	0.0543 (8)	
H3A	0.152757	0.030205	0.243822	0.065*	
C4	0.1330 (5)	0.1370 (3)	0.0483 (5)	0.0588 (9)	
H4A	0.086569	0.091344	−0.048297	0.071*	
C5	0.1641 (5)	0.2419 (3)	0.0174 (4)	0.0558 (8)	
H5A	0.139312	0.266660	−0.100398	0.067*	
C6	0.2322 (4)	0.3105 (2)	0.1614 (4)	0.0426 (7)	
C7	0.3439 (5)	0.4963 (2)	0.6318 (4)	0.0413 (7)	
H7A	0.421014	0.463484	0.734710	0.050*	
C8	0.2957 (4)	0.6082 (2)	0.6376 (3)	0.0359 (6)	
C9	0.2219 (5)	0.6675 (2)	0.4811 (4)	0.0433 (7)	
H9A	0.201931	0.635668	0.368765	0.052*	
C10	0.1784 (5)	0.7724 (2)	0.4908 (4)	0.0512 (8)	
H10A	0.131772	0.810787	0.384850	0.061*	
C11	0.2029 (5)	0.8214 (2)	0.6557 (4)	0.0475 (7)	
H11A	0.169393	0.891678	0.662994	0.057*	
C12	0.2790 (4)	0.7623 (2)	0.8092 (4)	0.0387 (6)	
C13	0.3274 (4)	0.6576 (2)	0.8048 (4)	0.0375 (6)	
H13A	0.380225	0.620594	0.911236	0.045*	

### Atomic displacement parameters (Å^2^)

**Table:**

	*U*^11^	*U*^22^	*U*^33^	*U*^12^	*U*^13^	*U*^23^
Cl1	0.0720 (6)	0.0433 (4)	0.0608 (5)	0.0025 (4)	0.0200 (4)	0.0100 (4)
Cl2	0.0988 (8)	0.0470 (4)	0.0610 (5)	0.0050 (5)	0.0339 (5)	0.0121 (4)
O1	0.122 (2)	0.0705 (17)	0.0406 (13)	0.0047 (16)	0.0118 (14)	−0.0139 (12)
O2	0.106 (2)	0.0565 (16)	0.0844 (19)	0.0181 (15)	0.0135 (16)	−0.0350 (14)
N1	0.0622 (17)	0.0308 (12)	0.0418 (13)	0.0081 (10)	0.0060 (11)	−0.0040 (9)
N2	0.0457 (13)	0.0285 (10)	0.0451 (12)	0.0019 (11)	0.0128 (10)	−0.0042 (10)
N3	0.0580 (18)	0.0489 (15)	0.0529 (16)	−0.0065 (13)	0.0163 (14)	−0.0188 (13)
C1	0.0341 (15)	0.0321 (12)	0.0434 (14)	0.0045 (11)	0.0110 (12)	−0.0048 (10)
C2	0.0420 (17)	0.0315 (13)	0.0513 (17)	0.0049 (12)	0.0153 (13)	−0.0011 (11)
C3	0.0538 (19)	0.0350 (16)	0.073 (2)	0.0013 (14)	0.0155 (16)	−0.0127 (15)
C4	0.059 (2)	0.055 (2)	0.0569 (19)	0.0011 (16)	0.0081 (15)	−0.0228 (15)
C5	0.058 (2)	0.066 (2)	0.0420 (17)	0.0124 (17)	0.0099 (15)	−0.0063 (14)
C6	0.0452 (18)	0.0368 (14)	0.0477 (16)	0.0057 (13)	0.0158 (14)	−0.0013 (13)
C7	0.0522 (19)	0.0326 (14)	0.0385 (15)	0.0020 (12)	0.0116 (13)	−0.0017 (11)
C8	0.0366 (14)	0.0336 (13)	0.0379 (13)	−0.0044 (12)	0.0108 (11)	−0.0043 (12)
C9	0.0506 (19)	0.0418 (16)	0.0354 (15)	−0.0022 (13)	0.0080 (13)	−0.0064 (11)
C10	0.059 (2)	0.0423 (16)	0.0455 (18)	0.0014 (15)	0.0033 (15)	0.0060 (13)
C11	0.0504 (19)	0.0295 (13)	0.0594 (19)	0.0028 (13)	0.0097 (15)	−0.0045 (13)
C12	0.0380 (15)	0.0361 (14)	0.0417 (16)	−0.0054 (12)	0.0104 (12)	−0.0109 (11)
C13	0.0422 (16)	0.0355 (13)	0.0355 (14)	−0.0025 (11)	0.0119 (12)	−0.0009 (10)

### Geometric parameters (Å, º)

**Table:**

Cl1—C2	1.739 (3)	C4—H4A	0.9300
Cl2—C6	1.740 (3)	C5—C6	1.389 (4)
O1—N3	1.215 (4)	C5—H5A	0.9300
O2—N3	1.220 (4)	C7—C8	1.467 (4)
N1—N2	1.367 (3)	C7—H7A	0.9300
N1—C1	1.387 (3)	C8—C13	1.393 (3)
N1—H1N	0.9500	C8—C9	1.396 (4)
N2—C7	1.275 (3)	C9—C10	1.375 (4)
N3—C12	1.469 (3)	C9—H9A	0.9300
C1—C6	1.392 (4)	C10—C11	1.382 (4)
C1—C2	1.404 (4)	C10—H10A	0.9300
C2—C3	1.379 (4)	C11—C12	1.379 (4)
C3—C4	1.369 (5)	C11—H11A	0.9300
C3—H3A	0.9300	C12—C13	1.378 (4)
C4—C5	1.383 (5)	C13—H13A	0.9300
			
N2—N1—C1	120.7 (2)	C5—C6—Cl2	116.6 (2)
N2—N1—H1N	118.5	C1—C6—Cl2	121.8 (2)
C1—N1—H1N	119.7	N2—C7—C8	120.4 (3)
C7—N2—N1	117.0 (2)	N2—C7—H7A	119.8
O1—N3—O2	122.6 (3)	C8—C7—H7A	119.8
O1—N3—C12	119.0 (3)	C13—C8—C9	118.7 (2)
O2—N3—C12	118.5 (3)	C13—C8—C7	119.0 (2)
N1—C1—C6	124.6 (2)	C9—C8—C7	122.2 (2)
N1—C1—C2	119.2 (3)	C10—C9—C8	120.9 (3)
C6—C1—C2	116.1 (2)	C10—C9—H9A	119.5
C3—C2—C1	122.5 (3)	C8—C9—H9A	119.5
C3—C2—Cl1	118.4 (2)	C9—C10—C11	120.9 (3)
C1—C2—Cl1	119.0 (2)	C9—C10—H10A	119.6
C4—C3—C2	119.9 (3)	C11—C10—H10A	119.6
C4—C3—H3A	120.1	C12—C11—C10	117.5 (3)
C2—C3—H3A	120.1	C12—C11—H11A	121.2
C3—C4—C5	119.6 (3)	C10—C11—H11A	121.2
C3—C4—H4A	120.2	C13—C12—C11	123.2 (2)
C5—C4—H4A	120.2	C13—C12—N3	117.7 (3)
C4—C5—C6	120.3 (3)	C11—C12—N3	119.1 (2)
C4—C5—H5A	119.8	C12—C13—C8	118.7 (2)
C6—C5—H5A	119.8	C12—C13—H13A	120.7
C5—C6—C1	121.5 (3)	C8—C13—H13A	120.7
			
C1—N1—N2—C7	177.3 (3)	N1—N2—C7—C8	178.2 (2)
N2—N1—C1—C6	35.6 (4)	N2—C7—C8—C13	165.5 (3)
N2—N1—C1—C2	−147.1 (3)	N2—C7—C8—C9	−15.6 (4)
N1—C1—C2—C3	−179.0 (3)	C13—C8—C9—C10	−1.0 (4)
C6—C1—C2—C3	−1.5 (4)	C7—C8—C9—C10	−179.9 (3)
N1—C1—C2—Cl1	−0.1 (4)	C8—C9—C10—C11	−1.3 (5)
C6—C1—C2—Cl1	177.4 (2)	C9—C10—C11—C12	2.3 (5)
C1—C2—C3—C4	−0.2 (5)	C10—C11—C12—C13	−1.2 (4)
Cl1—C2—C3—C4	−179.1 (3)	C10—C11—C12—N3	179.1 (3)
C2—C3—C4—C5	1.2 (5)	O1—N3—C12—C13	6.2 (4)
C3—C4—C5—C6	−0.4 (5)	O2—N3—C12—C13	−174.4 (3)
C4—C5—C6—C1	−1.4 (5)	O1—N3—C12—C11	−174.0 (3)
C4—C5—C6—Cl2	175.0 (3)	O2—N3—C12—C11	5.4 (4)
N1—C1—C6—C5	179.6 (3)	C11—C12—C13—C8	−1.0 (4)
C2—C1—C6—C5	2.3 (4)	N3—C12—C13—C8	178.8 (3)
N1—C1—C6—Cl2	3.4 (4)	C9—C8—C13—C12	2.0 (4)
C2—C1—C6—Cl2	−173.9 (2)	C7—C8—C13—C12	−179.0 (3)

### Hydrogen-bond geometry (Å, º)

**Table:**

*D*—H···*A*	*D*—H	H···*A*	*D*···*A*	*D*—H···*A*
N1—H1*N*···Cl1	0.95	2.54	2.940 (2)	106
N1—H1*N*···O1^i^	0.95	2.31	3.243 (3)	168

Symmetry code: (i) −*x*+1, *y*−1/2, −*z*+2.
